# Managing Lymphoma with Non-FDG Radiotracers: Current Clinical and Preclinical Applications

**DOI:** 10.1155/2013/626910

**Published:** 2013-06-06

**Authors:** Fan-Lin Kong, Richard J. Ford, David J. Yang

**Affiliations:** ^1^Department of Experimental Diagnostic Imaging, Box 59, The University of TX MD Anderson Cancer Center, 1515 Holcombe Boulevard, Houston, TX 77030, USA; ^2^Department of Hematopathology, The University of Texas MD Anderson Cancer Center, 1515 Holcombe Boulevard, Houston, TX 77030, USA

## Abstract

Nuclear medicine imaging modalities such as positron emission tomography (PET) and single-photon emission computed tomography (SPECT) have played a prominent role in lymphoma management. PET with [^18^F]Fluoro-2-deoxy-D-glucose (FDG) is the most commonly used tool for lymphoma imaging. However, FDG-PET has several limitations that give the false positive or false negative diagnosis of lymphoma. Therefore, development of new radiotracers with higher sensitivity, specificity, and different uptake mechanism is in great demand in the management of lymphoma. This paper reviews non-FDG radiopharmaceuticals that have been applied for PET and SPECT imaging in patients with different types of lymphoma, with attention to diagnosis, staging, therapy response assessment, and surveillance for disease relapse. In addition, we introduce three radiolabeled anti-CD20 antibodies for radioimmunotherapy, which is another important arm for lymphoma treatment and management. Finally, the relatively promising radiotracers that are currently under preclinical development are also discussed in this paper.

## 1. Introduction

According to the 2011 report from the National Cancer Institute's Surveillance, Epidemiology and End Results Program, an estimated total of 662,789 individuals in the US are living with, or in remission from lymphoma in 2011 [1]. About 75,190 people in the US are expected to be diagnosed with lymphoma in 2011, which include 8,830 cases of Hodgkin's lymphoma (HL) and 66,360 cases of non-Hodgkin's lymphoma (NHL). In fact, NHL is the seventh most common cancer in the US [1].

**Figure 1:**
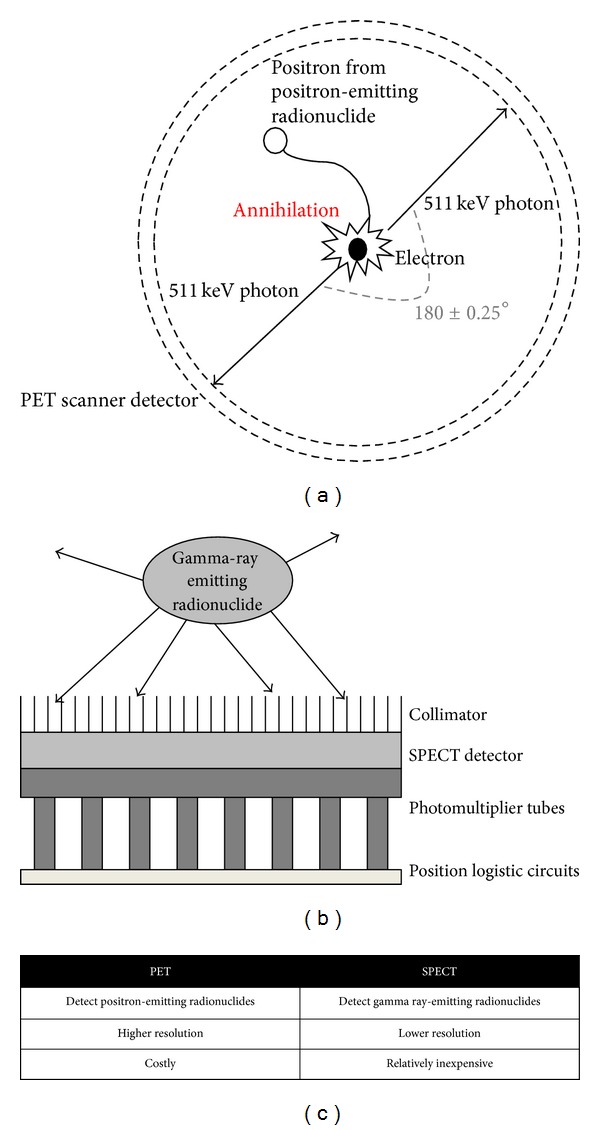
Comparison of positron emission tomography (PET) and single-photon emission computed tomography (SPECT). (a) Schematic representation of the principle behind PET, (b) schematic representation of the principle behind SPECT, and (c) comparison between PET and SPECT.

Lymphoma treatment and prognosis, especially for NHL, are heavily dependent on the disease type and staging. For instance, patients with stage I-II aggressive NHL respond to a short course of chemo/radiotherapy better than a full course of chemotherapy alone [2]. In early stage HL, 20%–30% of patients will relapse after mantle irradiation, which largely reflects inaccurate staging [3]. Therefore, it is extremely important to reach an accurate diagnosis, which can facilitate more precise staging and prognostic estimations, as well as evaluation of response to therapy. The major imaging modalities utilized in lymphoma are divided into two general types: the anatomic imaging modalities, such as computed tomography (CT) and magnetic resonance imaging (MRI), and the functional imaging modalities using ionizing radiation, such as positron emission tomography (PET) and single photon emission computed tomography (SPECT). CT and MRI can only provide limited information of lymphoma patients who have normal-sized lymph nodes, and they cannot differentiate tumor from lymphadenopathy, infection, hemorrhage, acute radiation pneumonitis, or radiation fibrosis [4, 5]. On the other hand, PET, SPECT, and their integration with CT can detect the biological alterations (increased glycolysis, DNA synthesis, amino acid transports, etc.) in tumor lesions in contrast to normal tissues and thus better distinguish viable tumor cells from necrotic cells or fibrosis. The principles of PET and SPECT imaging are both based on the detection of radiolabeled ligands; however, the radionuclides for these two modalities are quite different. PET detects the annihilation radiation emitted from a certain positron-emitting radionuclide, while SPECT detects the radionuclides that emit gamma-ray photons (Figure 1).

[^18^F]Fluoro-2-deoxy-D-glucose (FDG), an ^18^F-labeled glucose analogue, is the most commonly used radiotracer for PET imaging in lymphoma patients. In the most recent review of the literature search from 1999 to 2011 by Ansell and Armitage, FDG-PET is recommended for initial staging and re-staging at completion of therapy in patients with HL, diffuse large B-cell lymphoma (DLBCL), and follicular lymphoma (FL) [5]. However, its usage can be limited in cases of indolent diseases with low metabolic activity. In addition, FDG is not tumor specific and can also accumulate in inflammatory lesions such as tuberculosis, abscesses, and sarcoidosis [6–8]. FDG is not recommended for relapse monitoring and may not be reliable for initial staging and re-staging in patients with peripheral T-cell lymphoma and mantle cell lymphoma [9]. Furthermore, FDG-PET may not be definitive for interim response assessment in patients with HL and DLBCL, and detection of potential transformation sites. Therefore, development of new radiotracers with higher sensitivity, specificity, and different uptake mechanism is in great demand in the management of lymphoma. 

This paper first reviews the clinically used non-FDG radiopharmaceuticals for PET and SPECT imaging, respectively (Table 1), and discusses their advantages and limitations in staging, treatment monitoring, and relapse surveillance in lymphoma patients. The discussion also covers the current available radiopharmaceuticals for radioimmunotherapy (Table 2), which is another important option for lymphoma treatment and management. Lastly, a number of novel radiotracers that are currently under preclinical investigations have been focused on. 

## 2. Non-FDG Radiopharmaceuticals Used in Clinic

### 2.1. Non-FDG Radiopharmaceuticals for PET

#### 2.1.1. ^18^F-Fluorothymidine (FLT)

^18^F-fluorothymidine (FLT), a derivative of the cytostatic drug zidovudine, was developed as a proliferation imaging tracer in 1998 [10]. FLT is entrapped into cells during their S-phase, and its uptake correlates with the thymidine kinase-1 (TK-1) activity, which is a key enzyme for DNA synthesis and cellular growth [11]. FLT uptake in tumor cells is directly correlated with the proliferation marker Ki67 [12]. Buck et al. demonstrated that FLT could accurately discriminate between indolent and aggressive lymphoma in 34 patients with a cutoff SUV value of 3, and FLT uptake was significantly correlated with Ki67 immunohistochemical staining in biopsied tissues [6]. This important finding showed that FLT-PET might be superior to FDG-PET in lymphoma grading because the cutoff SUV for aggressive lymphoma using FDG is >13, and that for indolent lymphoma is <6, and about 45% of the patients remain in a grey zone [13]. More recently, Herrmann et al. conducted a pilot study using FLT-PET in Mantle cell lymphoma patients and showed a strong positive correlation between proliferation assessed with Ki67 staining or MIPI-Ki67 (a combined clinical and biologic score) and FLT uptake [14].

In addition, FLT-PET is considered as a promising sensitive tool for predicting response to treatment and survival in lymphoma patients. Although FDG-PET can identify patients who have an excellent prognosis after standard treatment, it has failed to accurately identify patients who would benefit from alternative treatment strategies or who should be included into clinical trials because of a dismal outcome with R-CHOP-like therapy [15]. In 2011, Herrmann et al. reported the largest clinical trial of FLT-PET in lymphoma patients and found FLT uptake as a negative predictor of response to R-CHOP treatment in 66 DLBCL patients. In this study, they also showed that FLT uptake was significantly correlated with the International Prognostic Index, which is a frequently used clinical tool to aid in predicting the prognosis of patients with aggressive NHL [16].

In respect to treatment monitoring and evaluation, FLT appears to be more accurate and specific than FDG, particularly in the setting of interim PET analyses. This is possibly because FDG uptake often occurs in chemo/radiation therapy-mediated inflammatory lesions besides neoplastic tissues, both of which demand more glucose uptake than other normal tissues. Herrmann et al. evaluated FLT-PET for assessing early response of high-grade NHL to rituximab immunotherapy combined with CHOP chemotherapy or CHOP alone and found that successful R-CHOP/CHOP treatment was associated with a decrease in FLT uptake even 2 days after administration of R-CHOP, whereas no reduction of FLT uptake after rituximab treatment alone, indicating no early antiproliferative effect of immunotherapy using rituximab [17]. Moreover, a significant difference in tumorous FLT uptake between patients in partial response and complete response was observed in the 14 patients receiving a PET scan early after chemotherapy completion (*n* = 8, 2 days after R-CHOP; *n* = 6, 7 days after R-CHOP/CHOP) [17].

#### 2.1.2. ^11^C-Methionine (MET)

^11^C-methionine (MET) is the most commonly used radiolabelled amino acid for lymphoma imaging. Methionine is essential for protein synthesis and conversion to the predominant biologic methyl group donor S-adenosylmethionine, and it involves polyamine synthesis and transsulfuration pathway [18]. MET accumulates strongly in most lymphomas, and it has low uptake in macrophages and nonneoplastic cells. MET uptake reflects increased amino acid uptake and protein synthesis and is positively related to cellular proliferation activity. Previously, Nuutinen et al. investigated whether MET uptake was associated with the histological grade of malignancy and survival in NHL and HL patients with newly diagnosed or recurrent lymphoma, and demonstrated that it was able to differentiate the high grade lymphomas from the low grade histotypes if using influx constant Ki instead of the traditional SUV calculation. In addition, they found that it was not feasible to use MET-PET for prediction of patient survival [19].

MET is preferable to FDG in some situations where FDG is inaccurate, for example, in hyperglycaemic patients [20]. Leskinen-Kallio et al. demonstrated that MET was superior to FDG in detecting intermediate- and low-grade lymphomas, and MET could accumulate strongly in all except one of the neoplastic lesions from 14 NHL patients [18]. 

The central nervous system (CNS) generally has high glucose consumption, which leads to high FDG uptake in the normal neuronal tissues and thus renders low contrast from tumors to normal tissues in the CNS. And yet, MET has demonstrated its effectiveness in detecting CNS lymphoma, which represents 6% of all intracranial neoplasms and 1% of all lymphomas [21–23]. In comparison to FDG, MET has lower uptake in normal brain, hence, has better contrast in visualizing tumor lesions. Ogawa et al. first performed MET-PET in 10 patients with histologically verified CNS lymphoma before and after radiation therapy [23]. They found that all tumors could clearly be defined by MET before treatment, and the uptake decreased markedly after radiation therapy. In addition, MET-PET could even demonstrate the residual tumor that was difficult to be detected on CT and MRI because of the influence of radiation therapy and surgical treatment. Kawase et al. showed that both MET and FDG could detect primary CNS lymphoma with 100% sensitivity in 13 immunocompetent patients [21]. However, Kawai et al. obtained a somehow contrary conclusion and pointed out that MET and FDG were both only useful in detecting the lesions with typical MRI findings, but not in the lesions with atypical MRI presentations such as disseminated, ring-like enhanced, or nonenhancing lesions [22]. 

### 2.2. Non-FDG Radiopharmaceuticals for SPECT

#### 2.2.1. ^67^Ga-Citrate

Among the single photon-emitting radiotracers, ^67^Ga-citrate has been considered a cornerstone in the evaluation of lymphoma for decades. ^67^Ga accumulates in viable lymphoma cells by binding to transferrin receptors, but typically, it is not taken up by fibrotic tissues. Although ^67^Ga imaging has been widely used in investigating treatment response, survival prediction, and diagnosis of recurrence after treatment [24], it has several limitations: (1) low spatial resolution, (2) low sensitivity for detection of hepatic and/or splenic lymphoma involvement due to the physiological uptake in these organs, and (3) low accumulation in low-grade lymphoma [25].

Many research groups have compared the performance of FDG-PET with ^67^Ga scintigraphy in lymphoma imaging. Their findings collectively suggested that FDG-PET is superior to ^67^Ga scintigraphy in pretreatment staging in both HL and NHL patients and can detect extra sites, especially the small regions of disease activity [26–28]. In addition, FDG-PET appears to be more sensitive in the followup of patients with de novo HL [27]. Fusion imaging with ^67^Ga-SPECT and CT is of significance in improving diagnosis by allowing precise localization of radiopharmaceutical uptake and detection of lesions not demonstrated by CT. In 2005, Palumbo et al. for the first time demonstrated that ^67^Ga-citrate performance could be improved by using SPECT/CT fusion imaging, suggesting that this modality could represent an alternative to PET [29]. They found that hybrid imaging provided additional data in 54.2% patients, thus leading oncologists to reconsider the therapeutic approach in 33.2% patients. Moreover, 9 more lesions below the diaphragm were detected by SPECT/CT as compared with SPECT alone. This is of particular interest because one limitation of  ^67^Ga scintigraphy is its restricted ability to identify subdiaphragmatic disease. However, the limitation of this study was that the authors only compared the results with SPECT alone but did not compare with FDG-PET. Further studies of comparison between FDG-PET or FDG-PET/CT and ^67^Ga-SPECT/CT would be of significant clinic interest. 

#### 2.2.2. Thallium-201 (^201^Tl)

Thallium-201 (^201^Tl) behaves biologically like potassium. Its tumor uptake is related to multiple factors such as blood flow, tumor type, tumor viability, vascular immaturity, increased cell membrane permeability, and activity of sodium-potassium adenosinetriphosphatase (Na-K-ATPase), non-energy-dependent cotransporter, and calcium ion channel [30]. Ando et al. demonstrated that ^201^Tl mainly accumulated in viable tumor tissues, less so in connective tissues, and barely in necrotic tumor tissues and inflammatory sites [31].

^201^Tl scintigraphy is valuable in evaluating chemo/radiotherapy treatment response because the activity of Na-K-ATPase in tumor cells decreases after treatment, and thus less ^201^Tl uptake should be observed. Haas et al. evaluated the usage of ^201^Tl in staging and monitoring treatment response after radiotherapy in FL patients [32]. They concluded that although ^201^Tl had limited value in staging FL patients, it was accurate in monitoring the responses of radiation treatment. If an FL patient with a positive ^201^Tl at diagnosis is treated by radiation, the treatment response can be reliably ascertained by ^201^Tl scintigraphy alone.

In comparison to ^67^Ga scintigraphy which is highly sensitive in high-grade lymphoma detection, ^201^Tl is more frequently utilized in imaging low-grade lymphomas [33]. In addition, ^201^Tl scintigraphy is more convenient than ^67^Ga scintigraphy because it can be performed immediately after injection. The optimal time of ^201^Tl scintigraphy is 3-4 hours after injection whereas that of ^67^Ga scintigraphy is 2 days due to the longer half-life of ^67^Ga [34]. For practical purposes, nevertheless, ^67^Ga and ^201^Tl scintigraphy should complement one another in the follow-up of indolent lymphoma. For instance, if a patient who used to be negative on ^67^Ga scintigraphy and positive on ^201^Tl converts to a positive status on ^67^Ga, it is likely that the indolent tumor has transformed to an aggressive pattern.

Furthermore, ^201^Tl brain SPECT has been successfully applied for differentiating CNS lymphoma from toxoplasmosis in patients with AIDS [35–37]. Lorberboym et al. demonstrated that the retention index of ^201^Tl in patients with lymphomas was significantly higher than that in patients with adenocarcinoma or nonmalignant lesions [36]. Moreover, Skiest et al. found that diagnostic accuracy of focal CNS lesions in patients with AIDS could be significantly improved with combining ^201^Tl brain SPECT with serum toxoplasma IgG [37].

#### 2.2.3. ^99m^Tc-Sestamibi and ^99m^Tc-Tetrofosmin

**Figure 2:**
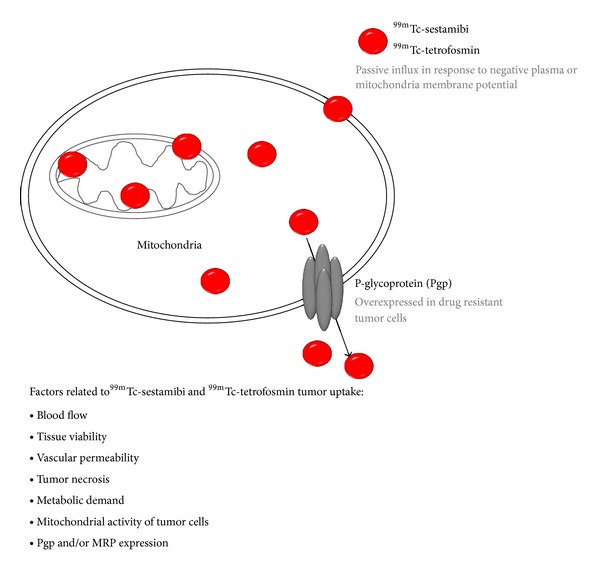
The uptake and efflux of  ^99m^Tc-sestamibi and ^99m^Tc-tetrofosmin in tumor cells.

^99m^Tc-sestamibi and ^99m^Tc-tetrofosmin, which were originally developed as myocardial perfusion agents, have been frequently used as predictors of chemotherapeutic response in lymphoma patients [38]. These agents preferentially accumulate in the mitochondria of malignant cells due to the higher metabolic rate, and thus the higher transmembrane electrical potentials generated across the membrane bilayers in these cells when compared with normal cells (Figure 2). These two small lipophilic monovalent cations are both transport substrates for the intraextracellular efflux pump of the transmembrane P-glycoprotein (Pgp). Of note, Pgp is encoded by the multidrug-resistance gene (*MDR1*) that is overexpressed in some drug resistant lymphoma cells [39, 40]. The net cellular accumulation of ^99m^Tc-sestamibi has been shown to be inversely proportional to the level of Pgp expression *in vitro* [41, 42]. Therefore, the uptake, washout rate, and retention of ^99m^Tc-sestamibi and ^99m^Tc-tetrofosmin can aid in identification of drug resistance and provide prognostic information [43]. In other words, the patients with negative or decreased radiotracer activity tend to have unfavorable response to chemotherapy compared to those with prominent radiotracer accumulation irrespective of lymphoma types. For instance, Song et al. demonstrated that the slow tumor clearance of ^99m^Tc-sestamibi could predict a good response to chemotherapy, and difference in ^99m^Tc-sestamibi clearance distinguishes responding and nonresponding tumors in the early course of chemotherapy in diffuse large B-cell and peripheral T-cell lymphoma patients [44]. Kao et al. found that patients with a good chemotherapy response had positive ^99m^Tc-sestamibi results and negative Pgp and MRP (multidrug resistance associated protein) expression before treatment, while patients with a poor response had negative ^99m^Tc-sestamibi results and positive Pgp and MRP expression [45]. Liang et al. concluded that ^99m^Tc-tetrofosmin uptake, in inverse correlation with Pgp or MRP expression levels, could accurately predict chemotherapy response in 25 lymphoma patients [46].

In another study, Lazarowski et al. demonstrated that the patients with low grade lymphoma had the strongest correlation between ^99m^Tc-sestamibi uptake and chemosensitivity, while patients with HL had an indefinable correlation [47]. In addition, the later scan (180 min after injection) could provide more accurate prediction of chemoresistance than early scan (30 min after injection) [47]. In general, factors related to ^99m^Tc-sestamibi and ^99m^Tc-tetrofosmin uptake in tumors are blood flow, tissue viability, vascular permeability, tumor necrosis, metabolic demand, tumor mitochondrial activity, and Pgp and/or MRP expression in tumor tissues [44].

When comparing these two radiotracers, ^99m^Tc-tetrofosmin can be easily labeled with ^99m^Tc at room temperature without heating; hence, it is more convenient than ^99m^Tc-sestamibi in clinical practice [48]. Although ^99m^Tc-tetrofosmin has lower uptake in lymphoma cell lines [49], it undergoes more rapid clearance from the plasma and background structures when compared to ^99m^Tc-sestamibi. Current clinical investigations have demonstrated that both radiotracers are competent for prediction of chemotherapy response; however, no study has ever compared these two radiotracers to each other in lymphoma patients. The optimal imaging time-point for both tracers is 3-4 hours after injection. It should to be noted that these radiotracers are not ideal in investigating the infradiaphragmatic regions because both radiotracers are eliminated by the biliary-intestinal route [50].

#### 2.2.4. Somatostatin Receptor Scintigraphy

Somatostatin receptor scintigraphy (SRS) using ^111^In-labeled octreotide has been frequently applied in neuroendocrine tumor imaging. It has also been successfully used in detecting somatostatin receptor-expressing lymphomas such as mucosa associated lymphoid tissue- (MALT-) type lymphoma. Octreotide is a synthetic somatostatin analogue that is available as Octreoscan (Mallinckrodt Inc., MO), in which the gamma-emitting radioisotope ^111^In has been chelated with octreotide via chelator DTPA. The overall sensitivity of SRS with Octreoscan for HL is 95%–100%, and for NHL is around 80% [51]. Nevertheless, the sensitivity is decreased in detection of abdominal lesions, and the specificity of this technique is relatively low due to the variable expression of specific somatostatin receptor subtypes in lymphomas. For instance, Valencak et al. did not recommend the use of SRS for routine staging of primary cutaneous T-cell and B-cell lymphoma with Octreoscan based on the unfavorable outcome of a study involving 22 patients. In this study, only 4 out of 15 patients with cutaneous T-cell lymphoma and 3 out of 7 patients with B-cell lymphoma could be detected by Octreoscan [52].

Although SRS with Octreoscan does not seem to have a significant impact on patients with lymphomas for diagnostic purposes, it appears to be an excellent tool for staging and noninvasive therapy-monitoring in extragastric MALT-type lymphomas. In a study of 30 patients with extragastric manifestations of MALT-type lymphoma, Raderer et al. found that Octreoscan is superior to conventional imaging techniques in terms of noninvasive evaluation of treatment efficacy [53]. In addition, it allows distinction between gastric versus extragastric origin of the MALT-type lymphoma in patients with lesions located outside the GI tract. While no positive scans were obtained in patients with gastric MALT-type lymphomas irrespective of size and stage, excellent visualization of lymphomas originating in extragastric sites could be achieved using Octreoscan [54]. Furthermore, it was suggested that Octreoscan may identify patients suitable for therapy with labeled or unlabeled somatostatin analogues; however, no clinical studies have yet supported this idea. In another study, Li et al. compared ^67^Ga scintigraphy results with those obtained by ^111^In-DOTA-_D_Phe^1^-Tyr^3^-octreotide and ^111^In-DOTA-lanreotide scintigraphy, which were two octreotide analogues, in 18 patients with proven MALT-type lymphoma [55]. Although there were no statistically significant differences in patient- and site-related sensitivities among three radiotracers, the sensitivity of ^111^In-labeled compounds tended to be superior to that of  ^67^Ga scintigraphy for infradiaphragmatic involvement but inferior for supradiaphragmatic lesions.

## 3. Radiopharmaceuticals for Radioimmunotherapy Management

Low-grade lymphomas are refractory to most treatments, and each subsequent treatment is less effective. Radioimmunotherapy with a tumor-specific antibody conjugated to a beta-emitting radioisotope will deliver radiation not only to tumor cells that bind to the antibody, but also, due to a cross-fire effect, to neighboring tumor cells that are inaccessible to the antibody or with insufficient target-antigen expression. At present, the most successful radioimmunotherapy agents for lymphomas are radiolabeled anti-CD20 monoclonal antibodies such as ^90^Y-labeled Zevalin (Ibritumomab Tiuxetan) and ^131^I-labeled Bexxar (Tositumomab) [56]. CD20 is a transmembrane protein that acts as a calcium channel and plays an important role in cell cycle progression and differentiation of normal and malignant B-cells. CD20 is present in the lymphoma cells in more than 90% patients with B-cell NHL, and it is not expressed on uncommitted hematopoietic precursor stem cells. When anti-CD20 antibodies bind to the antigen, they induce apoptosis, antibody-dependent cellular cytotoxicity, and complement-dependent cytotoxicity in lymphoma cells [57]. Therefore, CD20 is a suitable target for imaging and treatment of NHL. Clinical practices have indicated that radioimmunotherapy using Zevalin and Bexxar is an effective and safe adjunctive treatment for patients with NHL refractory/relapsed to conventional treatment [56–61]. Next we introduce three radiolabeled anti-CD20 antibodies: Zevalin, Bexxar, and ^131^I-rituximab. Zevalin and Bexxar have been approved by FDA while ^131^I-rituximab is still under clinical trial. 

### 3.1. Radiolabeled Zevalin

Zevalin (Ibritumomab Tiuxetan) is a murine IgG_1a_ kappa monoclonal antibody that binds specifically to the CD20 antigen on normal and malignant B-lymphocytes [62]. It is the first radioimmunoconjugate approved by US FDA in 2002 and Europe in 2004 for radioimmunodiagnosis (^111^In-Zevalin) or radioimmunotherapy (^90^Y-Zevalin) in patients with follicular NHL refractory to rituximab. By using the chelator Tiuxetan (MX-DTPA), ^111^In (gamma emitter; *t*_1/2_ = 67.2 hrs) and ^90^Y (pure beta emitter; *t*_1/2_ = 64 hrs) can be stably linked to Ibritumomab for imaging and treatment, respectively. In fact, ^111^In-Zevalin scan is required for ^90^Y-Zevalin therapy by US FDA to measure organ-specific accumulation and determine whether pretreatment dosimetry is necessary. In the FDA approved protocol, 250 mg/m^2^ unlabeled rituximab is given to the patient 48–72 hrs prior to performing the ^111^In-Zevalin scan (5 mCi/1.6 mg) in order to minimize uptake of ^111^In-Zevalin in normal tissues and blood mononuclear cells [63]. The patients with relapsed low-grade, follicular, or transformed B-cell NHL can be treated in an outpatient setting with a reported response rate of 74% with no significant adverse side effects. Interestingly, Iagaru et al. observed an inverse correlation between the extent of disease visible on ^111^In-Zevalin scans and the response to ^90^Y-Zevalin in 28 NHL patients, with a higher rate of complete response observed to ^90^Y-Zevalin in patients with negative ^111^In-Zevalin findings and a higher rate of disease progression noted despite therapy in patients with positive ^111^In-Zevalin findings [64]. However, these findings need to be confirmed in a larger prospective trial. In addition to the aforementioned NHLs, Iwamoto et al. demonstrated the feasibility of ^111^In/^90^Y-Zevalin in treatment management in 6 patients with primary CNS lymphoma in a pilot study [65]. They showed that ^111^In-Zevalin could penetrate into CNS lymphoma at higher levels than into normal brain; however, ^90^Y-Zevalin administration with a 33% response rate did not represent an ideal treatment to patients.

As described previously, ^111^In-Zevalin is required for radioimmunodiagnosis in the United States, but not most of the European countries. In the recent paper by Otte, he discussed and listed the reasons for not requiring ^111^In-Zevalin before radioimmunotherapy as follows: (1) ^90^Y-Zevalin dose is only based on patient's body weight and platelet count [66]; (2) the rate of truly altered biodistribution is very rare, with only 6 out of 953 patients (0.6%) according to the report by Conti et al [67]; (3) the correlation between ^111^In-Zevalin and ^90^Y-Zevalin distribution is only partly correct because partial disassociation of ^90^Y and ^111^In from the immunoconjugate may occur *in vivo*, and the free ^90^Y deposits on bone surfaces while free ^111^In preferentially goes to the germ cells of testes [68]; (4) the dosimetry study in clinical trials has shown no correlation between toxicity and the absorbed dose, and all absorbed dosages remained well below the thresholds of 4 Gy for the bone marrow and 20 Gy for other organs [69]. 

Perk et al. first radiolabeled Zevalin with a PET radioisotope zirconium-89 (^89^Zr; *t*_1/2_ = 78.4 hrs) in order to quantify ^90^Y-Zevalin biodistribution and dosimetry more accurately for high-dose radioimmunotherapy [70]. Because Tiuxetan does not bind to the four-valent ^89^Zr, the authors introduced N-succinyldesferal (N-sucDf) as a second chelator to Zevalin. Recently, Rizvi et al. reported a pilot study showing that pretherapy PET scan with ^89^Zr-Zevalin could be used to accurately predict radiation dosimetry for treatment with ^90^Y-Zevalin in 7 patients with relapsed B-cell NHL scheduled for autologous stem cell transplantation [71]. However, the highest absorbed dose of ^89^Zr-Zevalin was found in liver, but not in spleen as that of ^111^In-Zevalin, suggesting a different biodistribution between two radiotracers [72].

### 3.2. ^131^I- Tositumomab (Bexxar)

Tositumomab is a murine IgG2a anti-CD20 monoclonal antibody, and its ^131^I-labeled form has been approved in US in 2003 for the treatment of patients with CD20 positive follicular NHL, with and without transformation, whose disease is refractory to rituximab and has relapsed following chemotherapy [73]. Different from the weight-based dosing ^90^Y-Zevalin, the gamma photons emitted by ^131^I allow for applications in planar or SPECT imaging, while the comparatively long half-life (8.01 days) of ^131^I confers patient-specific calculation of the radioactivity that needs be administered to achieve desired therapeutic effects [74]. In addition, ^131^I has a tighter distribution of tumor-absorbing doses of radiation for a given tumor site and is predicted to be more efficacious in the treatment of lung nodules, particularly those with radii less than 2 cm, presumably due to the shorter path length of ^131^I. This finding may be of particular relevance to small tumor foci near normal tissues, if it can be extrapolated beyond lungs [75].

Because ^131^I-labeled antibody clearance varies significantly among patients, prescription of ^131^I- Tositumomab (product name Bexxar) activity must be based on a calculated total-body dose derived from quantitative whole-body imaging. Briefly, patients first receive an infusion of unlabeled Tositumomab to optimize the biodistribution and tumor-targeting of Bexxar. After 1 hr, Bexxar (5 mCi) is administered, and patients then undergo dosimetric whole-body imaging on at least three occasions during the following week [76]. This approach is necessary to ensure that a therapeutic dose is delivered and to reduce the risk of treatment-related toxicity. Once the minimum required activity being calculated, patient receives a second infusion of unlabeled Tositumomab, followed by the therapeutic radiolabeled Bexxar, usually 1-2 weeks after the dosimetric study [77]. The maximum tolerated total body dose has been established at 75 cGy in patients with adequate bone marrow reserves and less than 25% bone marrow involvement by lymphoma, 65 cGy in patients with mild thrombocytopenia, and 45 cGy in patients who have received stem cell transplantation [77]. The optimal time to initially assess the response after Bexxar therapy remains unclear so far. And yet, Jacene et al. found that a response at 12 weeks after treatment correlated with long-term survival, and therefore they proposed this time point for initial treatment evaluation [74].

Iagaru et al. compared Bexxar with ^90^Y-Zevalin in the management of 67 patients with low-grade refractory or relapsed NHL [78]. Both treatments provided an effective and safe adjunctive therapeutic regimen for the patients; however, ^90^Y-Zevalin appeared to be more effective than Bexxar in terms of objective, complete, and partial responses, but with a higher frequency of adverse effects. Nevertheless, no statistical significance was obtained from this retrospective study due to small number of patients. Jacene et al. performed a similar study and concluded that both drugs were well tolerated, but Bexxar caused significantly less severe declines in platelet counts and therefore may be a more appropriate choice for patients with limited bone marrow reserve [74].

### 3.3. ^131^I-Rituximab

Rituximab is a chimeric IgG1 kappa anti-CD20 antibody that mediates complement- and antibody-dependent cytotoxicity *in vitro*. In fact, the introduction of rituximab has truly revolutionized the management of patients with B-cell NHL [79]. In addition to serving as a single agent as standard therapy for relapsed or refractory indolent NHL, rituximab has also been used in combination with CHOP chemotherapy (cyclophosphamide, doxorubicin, vincristine, and prednisone) in treatment of both indolent and aggressive NHLs.

Similar to Bexxar, rituximab can be radiolabeled with ^131^I through a relatively simple mAb radioiodination procedure without the requirement of chelators. ^131^I-rituximab, however, has a higher whole body radiation dose as well as mean biological and effective whole body half-life compared with Bexxar (85 hrs versus 56 hrs) [80–82]. In a physician-sponsored Phase II trial, Turner et al. found that ^131^I-rituximab was effective with an objective response rate (ORR) of 71% in 35 patients with a median followup of 14 months. Completed remission (CR) was achieved in 54% of the patients with median duration of 20 months. These results were similar to those of Bexxar (ORR: 71%, CR: 34%, median progression free survival: 12 months for all responders and 20 months for CR patients) [76]. In another pilot study in 7 mantle cell lymphoma patients who had relapsed after high-dose chemotherapy with autologous stem cell transplantation, Behr et al. performed the treatment with myeloablative doses of 261–495 mCi of ^131^I-rituximab and found that this high-dose therapy appeared to be associated with a high response rate. However, 5 of 7 patients developed hypothyroidism in this trial despite thyroid blocking, suggesting the moderate toxicity of myeloablative dose of ^131^I-rituximab [59]. Leahy and Turner reported the largest-to-date single-center routine clinical study with 142 consecutive patients who received ^131^I-rituximab radioimmunotherapy for low-grade, predominantly follicular, relapsed NHL in 10 years. Toxicity was limited to hematologic grade 4 neutropenia, the ORR was 67%, CR was 50%, and overall median survival was 32 months [61]. Taken together, the current data with nonmyeloablative and myeloablative treatment using ^131^I-rituximab clearly suggest that ^131^I-rituximab can achieve high ORR and CR rates in relapsed or refractory NHLs, and both the hematologic and nonhematologic toxicities are similar to Bexxar, as long as critical radiation doses of 75 cGy to the total body (for nonmyeloablative) or 2700 cGy to lung (for myeloablative) are not exceeded [83].

## 4. Non-FDG Radiotracers under Preclinical Development

### 4.1. ^124^I/^64^Cu-Labeled Anti-CD20 Minibody

The currently available immunoPET tracers are all based on *intact* antibodies, and as a result, days are required for the activity levels to drop sufficiently to allow acceptable target-to-background ratios [84]. Therefore, redesigning antibodies without compromising their specificity by reducing their size is of high interest from many research groups recently. Olafsen et al. developed ^124^I-labeled recombinant anti-CD20 rituximab fragment (scFv-C_H_3 dimer; 80 kDa) and evaluated it with PET/CT in mice bearing human CD20-expressing lymphoma. They found that this agent termed as radiolabeled “minibody” had exceptional high-contrast PET images with fast blood clearance *in vivo*. The average uptake in CD20-positive tumors was 12.9 ± 3.4%ID/g, and the ratio of CD20-positive tumor to CD20-negative tumor uptake was 7.0 ± 3.1 at 21 hr, suggesting its high specificity to target CD20. The authors also radiolabeled this minibody with ^64^Cu using chelator DOTA; however, its tumor uptake was not as good as that of ^124^I-labeled compound because of the residual activity in CD20-negative tumors and the liver [84].

### 4.2. ^18^F-Labeled Isatin Sulfonamide (^18^F-ICMT-11)

The capacity to evade apoptosis has been defined as one of the hallmarks of cancer. Therefore, monitoring tumor cell death induced by anticancer treatment can provide important predictive value in routine patient management or early clinical trials. During apoptosis, the activation of caspases, a family of cysteine proteases, induces the DNA degradation, which is the most noticeable and specific feature of apoptosis. And caspase-3, the central effector caspase, has been identified as an attractive biomarker of apoptosis. Isatin-based isatin 5-sulfonamide (ICMT-11) has been identified as a caspase inhibitor with subnanomolar affinity for caspase-3, high metabolic stability, and moderate lipophilicity [85]. Nguyen et al. radiolabeled ICMT-11 with ^18^F, and investigated its ability to image the drug-induced tumor apoptotic process in 38C13 murine B-cell lymphoma models. They demonstrated that ^18^F-ICMT-11 could bind to lymphoma *in vivo* by up to 2-fold at 24 hr posttreatment compared to vehicle treatment, and this increased signal activity was associated with increased apoptosis [86]. Although these preliminary results were very promising, more preclinical studies should be conducted to further warrant the usefulness of this radiotracer in imaging lymphoma.

### 4.3. Radiolabeled LLP2A Analogues

The integrins play a crucial role in lymphocyte homing and passing through the lymphocyte endothelial wall or to inflammation sites and may contribute to dissemination of NHL. One of its subtypes integrin *α*4*β*1, expressed in human hematopoietic cells, regulates lymphocyte trafficking. It is also found widely expressed in leukemia, lymphoma, melanoma, and sarcomas [87]. N-[[4-[[[(2-ethylphenyl) amino]carbonyl]amino]phenyl]acetyl]-N(epsilon)-6-[(2E)-1-oxo-3-(3-pyridinyl-2-propenyl)]-l-lysyl-l-2-aminohexanedioyl-(1-amino-1-cyclohexane)carboxamide (LLP2A) is a high-affinity, high-specificity peptidomimetic ligand that binds the activated *α*4*β*1 integrin [88]. Denardo et al. synthesized 7 different ^111^In- or ^64^Cu-labeled LLP2A derivatives and investigated their imaging potentials in Raji Burkitt lymphoma model (*α*4*β*1-positive) [88]. In this study, they concluded that the DOTA-chelated derivative ^111^In-LLP2A-DOTA exhibited the best tumor-to-nontumor ratios and showed the greatest potential for planar and SPECT imaging targeting the *α*4*β*1 in human lymphoma, and its ^64^Cu-labeled counterpart also demonstrated excellent tumor targeting competency in PET scans at both 4 hr and 24 hr, which warrants further investigations [88]. The same group recently reported two ^99m^Tc-labeled LLP2A derivatives ^99m^Tc-LLP2A-HYNIC and ^99m^Tc-LLP2A-HYNIC-PEG and evaluated their safety and imaging potentials in NHL-bearing dog model. Both tracers showed moderate tumor uptake over background, and tumor uptake in canine B-cell lymphoma decreased after chemotherapy [89].

## 5. Summary

In this review, we have discussed the clinically used non-FDG radiopharmaceuticals for PET and SPECT imaging of lymphoma, as well as the radiotracers currently under preclinical development. In addition, we have introduced several common radiopharmaceuticals for radioimmunotherapy, which is another crucial component for lymphoma treatment and management. One issue we would like to point out here is that most of the clinically used SPECT radiopharmaceuticals have not been evaluated with the hybrid SPECT/CT system, which can provide higher sensitivity and specificity through a better definition of organs involved in radiotracer uptake and determination of their precise relationship with adjacent structures [90]. Therefore, we suggest that the clinical studies of SPECT-based radiotracers should be validated by SPECT/CT in future. Furthermore, as noted, most of the radiopharmaceuticals we introduce here are designed based on a specific cancer biomarker such as increased DNA synthesis, upregulated amino acid transporter or somatostatin receptor expression, specific CD20 expression in B-cells, and cellular apoptosis. We believe that the advances in molecular biology of lymphoma research can lead to an increased understanding of the cancer biomarkers that contribute to lymphoma progression and thus warrant the development of more personalized and specific lymphoma-targeted imaging agents and treatments.

## Figures and Tables

**Table 1:** The clinically used radiopharmaceuticals for positron emission tomography (PET) and single-photon emission computed tomography (SPECT) imaging.

Modality	Radiopharmaceutical	Radionuclide	Half-life	Source	Uptake mechanism
PET	[^18^F]Fluorodeoxyglucose	^ 18^F	109 min	Cyclotron	Glucose transporter
	3′-Deoxy-3′-[^18^F]fluorothymidine	^ 18^F	109 min	Cyclotron	DNA replication
	^ 11^C-methionine	^ 11^C	20.4 min	Cyclotron	Amino acid transporter

SPECT	^ 67^Ga-citrate	^ 67^Ga	78.3 hr	Cyclotron	Transferrin receptor
	Thallium-201	^ 201^Tl	73.0 hr	Cyclotron	Multiple factors (i.e., Na-K-ATPase, non-energy-dependent cotransporter, etc.)
	^ 99m^Tc-sestamibi	^ 99m^Tc	6.0 hr	Generator	P-glycoprotein
	^ 99m^Tc-tetrofosmin	^ 99m^Tc	6.0 hr	Generator	P-glycoprotein
	^ 111^In-labeled Octreotide	^ 111^In	67.4 hr	Cyclotron	Somatostatin receptor

**Table 2:** The current available radiopharmaceuticals for radioimmunotherapy of lymphoma.

	^90^Y-Zevalin	^ 131^I-Bexxar	^ 131^I-Rituximab
Radioisotope	^90^Y (t_1/2_ = 2.67 days)	^ 131^I (*t*_1/2_ = 8.01 days)	^ 131^I (*t*_1/2_ = 8.01 days)
Anti-CD20 antibody	Ibritumomab tiuxetan	Tositumomab	Rituximab
Antibody type	Monoclonal murine	Monoclonal murine	Monoclonal chimeric
Predose injection	Unlabeled rituximab	Unlabeled tositumomab	Unlabeled rituximab
Pretherapy imaging	Yes (for biodistribution)	Yes (for dosimetry)	Yes (for dosimetry)
Pretherapy dose	^111^In-Zevalin (5 mCi)	^ 131^I-Bexxar (5 mCi)	^ 131^I-Rituximab (5 mCi)
Treatment dose	0.4 mCi/kg (up to 32 mCi)	75 cGy (whole body)	75 cGy (whole body)
